# Should Aggressive Surgical Local Control Be Attempted in All Patients with Metastatic or Pelvic Ewing's Sarcoma?

**DOI:** 10.1155/2012/953602

**Published:** 2012-03-04

**Authors:** Steven W. Thorpe, Kurt R. Weiss, Mark A. Goodman, Alma E. Heyl, Richard L. McGough

**Affiliations:** ^1^Department of Orthopaedic Surgery, University of Pittsburgh Medical Center, 3471 Fifth Avenue, Suite 1010, Pittsburgh, PA 15213, USA; ^2^Division of Musculoskeletal Oncology, Department of Orthopaedic Surgery, University of Pittsburgh Medical Center, UPMC Shadyside Medical Building, 5200 Centre Avenue, Suite 415, Pittsburgh, PA 15232, USA; ^3^Division of Musculoskeletal Oncology and Adult Reconstruction, Department of Orthopaedic Surgery, University of Pittsburgh Medical Center, UPMC Shadyside Medical Building, 5200 Centre Avenue, Suite 415, Pittsburgh, PA 15232, USA; ^4^Division of Musculoskeletal Oncology, Departments of Orthopaedic Surgery and Surgery, UPCI Sarcoma Service, University of Pittsburgh Medical Center, UPMC Shadyside Medical Building, 5200 Centre Avenue, Suite 415, Pittsburgh, PA 15232, USA

## Abstract

In previous reports, patients with Ewing's sarcoma received radiation therapy (XRT) for definitive local control because metastatic disease and pelvic location were thought to preclude aggressive local treatment. We sought to determine if single-site metastatic disease should be treated differently from multicentric-metastatic disease. We also wanted to reinvestigate the impact of XRT, pelvic location, and local recurrence on outcomes. Our results demonstrated a significant difference in overall survival (OS) between patients with either localized disease or a single-metastatic site and patients with multicentric-metastatic disease (*P* = 0.004). Local control was also found to be an independent predictor of outcomes as demonstrated by a significant difference in OS between those with and without local recurrence (*P* = 0.001). Axial and pelvic location did not predict a decreased OS. Based on these results, we concluded that pelvic location and the diagnosis of metastatic disease at diagnosis should not preclude aggressive local control, except in cases of multicentric-metastatic disease.

## 1. Introduction

Ewing's sarcoma (EWS) is the second most common primary bone tumor in children and adolescents [1–6], representing 3% of all pediatric malignancies [2, 5, 7]. Most arise from bone, but extraosseous EWS may occur [2]. Stage and tumor size have consistently been shown to be independent predictors of survival [8–12]. Additional factors traditionally thought to be prognostic of decreased survival are pelvic location, advanced age, and histological response to chemotherapy [2, 10, 13]. However, with improvement in treatment protocols and chemotherapy regimens, these factors deserve reinvestigation [2].

Despite advances in chemotherapy protocols, survival rates are consistently in the 54–68% range [8, 9, 12, 14, 15]. This plateau in the improvement of outcomes has been frustrating despite chemotherapy trials, new regimens, and dose intensification [5, 7, 14, 16]. Therefore, it is important to reinvestigate the impact of local control on overall survival. Previous studies, in which up to 80–90% of patients received radiation therapy (XRT) for local control with or without surgery, have shown that XRT alone for local control is associated with poor outcomes [8, 12, 17]. Many patients received XRT for local control because metastatic disease and pelvic location were thought to preclude aggressive local treatment [7, 14, 18–20].

The primary goal of this study was to investigate the clinical results of Ewing's sarcoma treated at our institution, where a smaller percentage of patients have received radiation for local control compared to previous studies. Our goal was to determine (1) if axial tumors have worse outcomes than appendicular tumors, (2) if there is a survival difference between multicentric metastatic EWS and metastases to a single location, (3) the efficacy of XRT for local control in terms of survival and complications, and (4) if there is a survival difference between osseous and extraosseous EWS.

## 2. Methods

After Institutional Review Board approval, we performed a retrospective review of all patients with the diagnosis of EWS treated at our tertiary musculoskeletal oncology center from 1981 to 2009. We reviewed patients' medical records for surgical reports, radiographic studies, and pathological results in order to determine primary location, AJCC stage at diagnosis, neoadjuvant treatment, primary local treatment, adjuvant treatments, local recurrence, late-metastatic disease, and late complications. For those patients that underwent surgical resection of a primary lesion, margins were described as negative or positive on the basis of review of pathologic analysis, and all complications were recorded.

Patients were excluded for insufficient followup (<2 years), except in the case of mortality prior to 2-year followup. Both osseous and extraosseous EWS were included for review. Length of followup, time to recurrence, and time to late metastases were calculated based on the date of diagnosis.

Forty-six patients treated between 1981 and 2009 were included with an average and median followup of 5.9 years and 3.5 years (range 0.2–29.8 years), respectively.

Kaplan-Meier survival curves were created for overall survival as well as event free survival. Log Rank (Cox-Mantel) test was used to determine statistical significance between survival curves. Multivariate Cox Regression was used to determine independent covariates. Chi-square analysis was used to determine correlation between variables. Statistical analyses were conducted with PASW Statistics 18 and 19 (IBM SPSS, IBM Corporation, Somers, NY).

## 3. Results

Forty-six patients treated between 1981 and 2009 (with 32/46 (70%) after 1998) were included with adequate followup or mortality. Only 5/46 (11%) patients were diagnosed prior to 1992. Patient characteristics are shown in Table 1. The average and median ages at diagnosis were 22 and 17 years (range 2–40 years), respectively, with 9 (20%) patients younger than 14 years of age. The male to female ratio was 1.6. Location was axial in 19/46 (41%) of primary lesions, with 11/46 (24%) pelvic lesions. Metastatic disease at the time of diagnosis was found in 13/42 (31%) of patients (AJCC stage at diagnosis was unknown for five patients), and 7/42 (17%) of patients presented with multicentric disease. Lung/mediastinum was the only location of single site metastatic disease in our series (Table 2). The treatment to the metastatic site in these six patients was radiation in three patients, none in two patients, and one patient underwent thoracotomy with resection. Neoadjuvant chemotherapy was given in 38 patients, five patients did not receive neoadjuvant chemotherapy prior to index surgical resection, and data was insufficient for three patients. One patient with a chest wall mass that was incompletely excised at an outside institution received neoadjuvant chemotherapy prior to complete chest wall resection at our institution. 

Surgical resection of the primary lesion was attempted in 38/45 (84%) patients. Twenty-six (74%) of these had negative margins (margin status was not available on three patients). Orthopaedic oncologists performed 32/38 (84%) resections. Limb salvage, and no amputations were performed at the index procedure. Patients that received surgical resection after radiation, resection for recurrence of EWS after XRT, or resection after biopsy were included as surgical resections. Seven patients did not undergo surgical resection, and one patient did not have sufficient primary treatment data. One patient with thoracic Ewing's underwent laminectomy for evolving lower extremity paralysis without goal of complete resection; this was not considered surgical treatment for primary location. One patient underwent chest wall resection after an incompletely resected chest wall tumor increased in size despite chemotherapy. This chest wall resection performed at our institution was considered the index procedure for our data. Nine patients underwent resection of pelvic EWS with negative margins achieved in five of nine (56%) (Table 3). Two of the patients with positive margins had recurrence or residual disease after XRT. The remaining two patients with positive margins had resections at an outside institution or by an orthopaedic spine surgeon. Five of eleven (33%) had died of disease at latest followup. Four of the five patients that died of disease had received XRT. The other patient that died of disease had multicentric metastatic disease at presentation and had an index surgery at an outside hospital without neoadjuvant or adjuvant chemotherapy.

Radiation was used for local control in only 10/43 (23%) of patients (three patients had insufficient data) (Table 4). Four patients who received radiation treatment for definitive local control had metastatic disease at diagnosis; two of these patients had multicentric-metastatic disease. Five patients underwent resection for residual disease or local recurrence after XRT. One patient that received preoperative XRT followed by resection eleven months after diagnosis, which showed residual tumor cells, was not considered a recurrence. Another patient underwent resection for recurrence of EWS that was found on bone scan more than two years after diagnosis, resulted in a few small areas of viable tumor. One patient developed a likely radiation-induced sarcoma (a high-grade retroperitoneal sarcoma with myofibroblastic phenotype and EWS translocation negative) at the site of radiation ten years after definitive local XRT. Complications, local recurrence, or radiation sarcoma occurred in seven of ten (70%) of patients treated with radiation. Chi-square analysis failed to show a significant correlation between local treatment with radiation and recurrence or complications.

Treatment failure was determined by event free survival (EFS). An event was defined as any local recurrence, late metastases, or death from disease. The local recurrence rate was 33% (15/46) and late metastases occurred in 45% (20/44) (Table 1). The average time from diagnosis till local recurrence was 1.65 years (Table 5). The one patient that developed radiation sarcoma was not included as a recurrence. The 5- and 10-year EFS was 52% and 38%. Age ≥ 14 (*P* = 0.021), multicentric metastatic disease at diagnosis (*P* = 0.002), and soft tissue Ewing's sarcoma (*P* = 0.020) exhibited significant effect on EFS according to Log Rank univariate analysis. Axial location, pelvic location, metastatic disease, local control with radiation, and margin status were not significant (Table 6). Only multicentric-metastatic disease (*P* = 0.007) and an extraosseous primary (*P* = 0.014) remained significant with Cox regression multivariate analysis (Table 7). Margin status was not included in multivariate analysis as it was not significant in univariate analysis, and two of seven patients with multicentric-metastatic disease would be excluded from multivariate analysis for lacking margin status at resection. Chi-square analyses did not show a significant correlation between pelvic location and tumor size or metastatic disease at presentation. 

**Figure 1:**
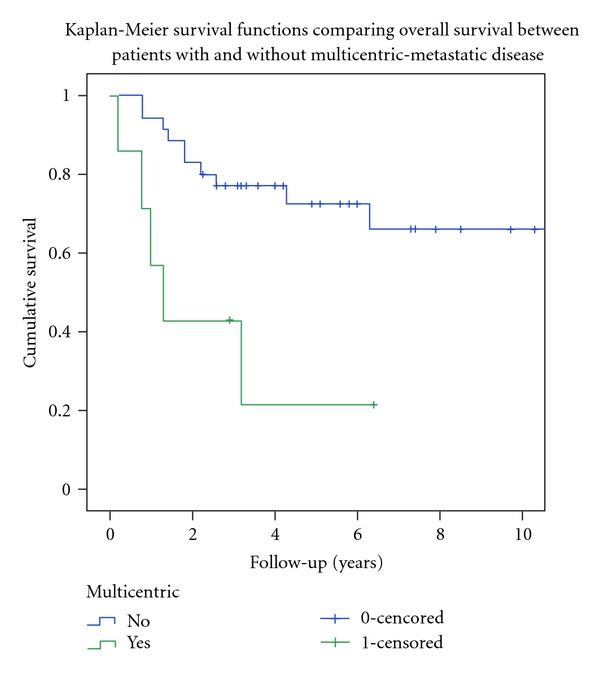
Kaplan-Meier overall survival curves comparing patients with and without multicentric-metastatic disease at diagnosis. Log Rank univariate analyses demonstrated significance (*P* = 0.004).

**Figure 2:**
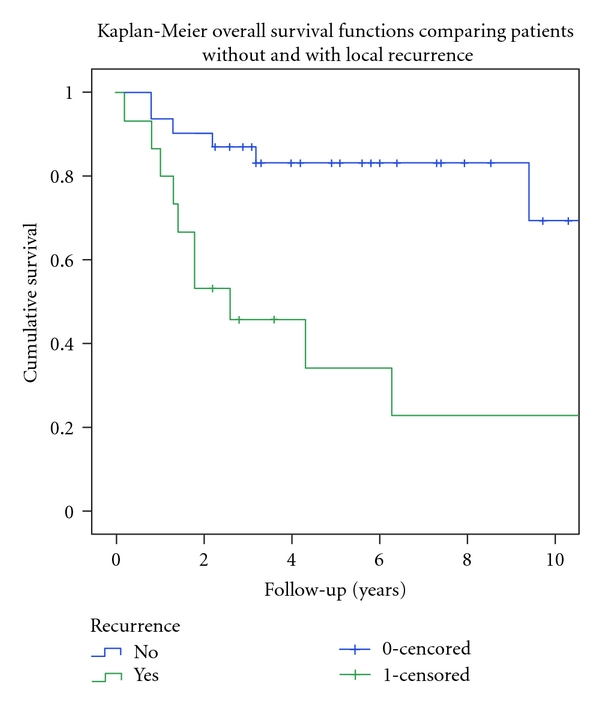
Kaplan-Meier overall survival curves comparing patients with and without local recurrence. Log Rank univariate analyses demonstrated significance (*P* = 0.001).

At latest followup, 50% (23/46) were alive without disease (AWOD), 13% (6/46) were alive with evidence of disease (AWED), 35% (16/46) had died of disease (DOD), and 2% (1/46) had died without disease (DWOD) (Table 1). The patient that died without evidence of disease, died second to radiation, for treatment of metastatic location, lung injury in the pediatric intensive care unit. Only one other patient with single site metastatic disease died, and this was at greater than six years after diagnosis. Four of the six (67%) of the patients with single site metastatic disease were AWOD at latest followup (Table 2). Overall survival calculation included both DOD and DWOD. The 5- and 10-year overall survival (OS) was 68% and 55%. The OS for the thirty-four patients with either localized disease or a single metastatic site was 73% at five years. The OS for the seven patients with multicentric-metastatic disease was significantly worse at 21% (*P* = 0.004) (Figure 1). The fifteen patients who sustained a local recurrence had a significantly worse 5-year OS (34% versus 83% for those without recurrence, *P* = 0.001) (Figure 2). Only one of fifteen patients (7%) with local recurrence was alive without evidence of disease at latest followup (Table 5). Age ≥ 14, axial location, pelvic primary, soft tissue primary, metastatic disease at diagnosis, local radiation, and margin status were not found to predict a poor outcome by Log Rank univariate analyses (Table 8). Cox regression multivariate analyses showed that multicentric-metastatic disease (*P* = 0.0291) and local recurrence (*P* = 0.0002) remained independent predictors of overall survival (Table 9). Margin status was not included in multivariate analysis as it was not significant in univariate analysis, and two of seven patients with multicentric metastatic disease would be excluded from multivariate analysis for lacking margin status at resection.

## 4. Discussion

Reports of overall 5-year survival for Ewing's sarcoma range from 57–77% [1, 5, 12, 14, 21]. Survival rates for localized disease are increased to 73–84% [3, 7, 22, 23]. Multiple large studies have consistently shown that tumor size and stage are important prognostic factors for overall survival with Ewing's sarcoma [8–10, 12]. In a more recent study with a 5-year EFS of 55.1% and a 5-year OS of 63.5%, Rodríguez-Galindo et al. confirmed that tumor size and stage were independent predictors of event free (EFS) and overall survival (OS) [8, 9]. Our study demonstrated similar 5-year EFS and OS with 52% and 68%, respectively, but we demonstrated that multicentric-metastatic disease and not the mere presence of metastatic disease was significant.

Location has consistently gained attention both as a prognostic variable and for local treatment determination. Axial and especially pelvic primary locations have been found to be prognostic of poorer survival with rates 18–51% [10, 14, 15, 20, 24]. Many of these studies included patients from older treatment eras with more of an emphasis on radiation. Shankar et al. in a study of 191 patients with localized Ewing's treated from 1987–1993 concluded that pelvic tumors had a worse outcome. In their study, 15% of localized disease of long bones received radiation only compared to 74% of localized disease of the pelvis with a 47% relapse rate for all localized pelvic disease regardless of treatment [23]. Only four of eleven (36%) of patients with pelvic disease in our series received radiation for local control (Table 3).

Rodriguez-Galindo et al. reported that tumor location has not shown the same significance with improved newer treatments [2]. Jürgens et al. and Wunder et al. found no relationship between pelvic site and event free survival [11, 25]. Even in studies of patients with only localized disease, the site of primary lesion was not prognostic, only the size, white blood cell count, and histological response to chemotherapy predicted EFS [3, 26]. More recent studies have supported that pelvic and/or axial locations alone were not associated with an increased local failure or decreased overall survival [8, 9].

These effects are due in part to an increased emphasis on surgical control and improved techniques. In a study dedicated to stage IIB pelvic Ewing's sarcoma, Yang et al. reported an increased overall survival of 51% with surgical resection compared to 18% without surgical resection [24]. Frassica et al. showed an increased OS of 5 years with resection over radiation alone (75% versus 25%) [20]. A pelvic 5-year OS rate of 64% and EFS of 48% in our series likely reflects improved local control methods with surgery and a later treatment era as none of our eleven pelvic tumors were diagnosed before 1992 and only four received prior radiation. Those patients that received radiation represented three of five patients that died of disease (Table 3).

Multiple studies and reports have repeatedly concluded that metastatic disease at the time of diagnosis is predictive of poorer outcomes [5, 8–10, 15]. The large European Intergroup study of 975 patients showed a significant difference in relapse free survival between patients with localized disease (55%) and those with metastatic disease at presentation (21%) [10], which is similar to our results with EFS 61% and 29% respectively. However, this result for us was not significant, probably due to study size limitations.

Multiple studies have demonstrated improved survival of pulmonary metastatic disease over extrapulmonary metastases [2, 8–10, 27]. Cotterill et al. demonstrated with univariate log rank analysis a significant difference between lung metastases and lung and bone metastatic disease [10]. However, multivariate analysis was not used to determine the independent effects on outcomes of metastatic location or single versus multicentric-metastatic disease. Wunder et al. found similar results to ours, with no significant difference in outcomes between localized and metastatic disease, but none of the patients had multifocal osseous lesions. Outcomes of patients with metastatic disease were linked to the degree of necrosis of the primary site [25]. We have shown that when multicentric-metastatic disease was the only independent predictor of both EFS and OS, and when patients with multicentric disease were selected out, the 5-year OS and EFS of patients without or a single metastatic location were 73% and 61%, respectively. According to Rodriguez-Galindo et al., there is a spectrum of metastatic disease from apparent localized disease with micrometastases to single-site metastatic disease to multicentric-metastatic disease [2]. Metastatic disease at presentation is important, but, perhaps, aggressive local control efforts should not be abandoned in cases of single-site metastatic disease. Local treatment to the site of metastatic disease in those patients with single-site metastatic disease should be further investigated in future studies. Questions that remain to be answered include, does XRT or surgical resection yield better outcomes and does size or number of lung metastases matter?

The role of local control has increasingly become important as a plateau has been reached with current chemotherapeutic regimens. Our local recurrence rate of 33% in an average of 1.65 years is in the wide range of published rates of 7–52% in 1.7–2.3 years [11, 14, 17, 20, 22, 25–30]. Recurrence rates for studies of only localized disease range 5–29% [3, 20, 31]. Local failure has been shown to be predicted by the treatment era, size of the primary tumor, and the type of local control [8, 9]. While margin status was not significant, our study demonstrated that local control is important for overall survival with local recurrence as an independent predictor of overall survival (*P* = 0.0002). Our Kaplan-Meier 5-year OS for patients with local recurrence was 34%  ±  14. Rodriguez-Galindo et al. reported a survival of 21.7 ± 7.8 for patients with local recurrence only [28]. Our survival after local recurrence does not separate out patients that had both local recurrence and late-distal metastatic disease, but multivariate analysis demonstrated that the presence of local recurrence independently predicts poor survival (Table 9). Our outcomes data for patients with local recurrence is likely even worse than represented by KM survival curve as only one of fifteen patients (7%) was alive without disease at latest followup (Table 5).

Radiation therapy alone as definitive local control for EWS has been found to correlate with increased local recurrence and a higher complication rate [2, 8, 10–12, 27, 28]. In previous studies, up to 90% of patients received radiation for primary local control [8, 17]. In the European Intergroup Cooperative Study, eight of nine patients that developed a secondary malignancy had received radiation [10]. Radiation has often been reserved for more difficult to resect locations such as the pelvis [7, 14, 18–20]. Radiation has also been preferred for local treatment in the presence of metastatic disease. Craft et al. demonstrated that 55% of patients with metastatic disease received radiation only for local control [14]. In a large study by Rodríguez-Galindo et al., radiation was found to be a significant factor for local control failure. Treatment era was also significant, but this likely also includes effects of increased radiation use in earlier treatment eras [8]. Rosito et al. showed that radiation alone for local treatment was associated with an increased local recurrence rate (15.3% versus 1.6%) [3]. Increased recurrence after local control with radiation alone could be attributed to the tumor's hypoxic “core” that is relatively radiation-resistant [17, 24]. In our study, radiation may not have been shown as a significant predictor of EFS or OS because only 10/43 (23%) received radiation. However, those patients that did receive radiation for local control did have a high complication and local recurrence rate of 70%.

With advances in resection and reconstruction techniques and technology, surgical resection for local control has increased. Increases in survival and EFS can be explained by more widespread utilization of surgical resection for local control [3, 12]. Lee et al. found a significant survival advantage with surgical resection compared with radiation alone [12]. Not only do the above studies indicate better local control and improved survival with surgical resection, but surgical resection of locally recurrent disease has been shown to increase survival rates after local recurrence [28]. Survival was even increased for those patients with positive margins after salvage resection [28], perhaps implicating a role in reducing tumor burden as an adjunct to aggressive salvage chemotherapy [32]. Similar results were shown in pelvic tumors [24].

Historically, extraskeletal Ewing's sarcoma has been treated similarly to skeletal lesions [32]. Studies have been conflicting regarding outcomes of these lesions [21, 27, 29, 33]. Pradhan et al. suggested that there was no survival difference between skeletal and extraskeletal disease (64% and 61% resp.) [27]. However, Applebaum et al. in review of the United States surveillance, epidemiology, and end results (SEER) database found a significant 5-year overall survival advantage for extraskeletal Ewing's over skeletal disease (69.7% versus 62.6%, *P* = 0.02) [33]. In the present study, extraskeletal lesions did not demonstrate a significantly worse overall survival, but both univariate and multivariate analyses identified extraosseous disease as a significant predictor of EFS as four of seven (57%) sustained local recurrence and five of seven (71%) sustained late-metastatic disease. An older study by Rud et al. from Mayo Clinic demonstrated local recurrence (46%) and late-metastatic disease (80%) rates similar to ours. Their study included patients treated between 1935 and 1985, but even when patients prior to 1970 were excluded, the 5-year OS was only 48% [29]. Our overall EFS and OS may have been worse than these other studies as only four of seven (57%) received chemotherapy prior to resection. Perhaps the conflicting outcomes for osseous and extraosseous outcomes warrants further cytologic and molecular testing of these two similar lesions.

## 5. Conclusion

Local control, marked by the effect of local recurrence, does play a significant role in overall survival. The mere presence of metastatic disease at diagnosis should not preclude aggressive local control, except perhaps in cases of multicentric metastatic disease. Pelvic disease offers unique anatomical challenges for resection and reconstruction, but we argue that it should be approached with aggressive surgical local control. Continued analyses of extraosseous lesions should be conducted to determine how their biology differs from Ewing's sarcoma of bone and if therapy should be tailored to individual tumor biology. 

## Figures and Tables

**Table 1:** Patient characteristics.

Variable (#)^†^	Number/percentage
Age (46)	
<14	9 (20%)
≥14	37 (80%)
Sex (46)	
Male	28 (61%)
Female	18 (39%)
Location (46)	
Appendicular	27 (59%)
Axial	19 (41%)
Pelvic (46)	
No	35 (76%)
Yes	11 (24%)
Bone or soft tissue (46)	
Bone	39 (85%)
Soft tissue	7 (15%)
Metastatic disease (42)	
No	29 (69%)
Yes	13 (31%)
Multicentric disease (42)	
No	35 (83%)
Yes	7 (17%)
Neoadjuvant chemotherapy (43)	
No	6 (14%)
Yes	37 (86%)
Local radiation^*δ*^ (43)	
No	33 (77%)
Yes	10 (23%)
Margins (35)	
Negative	26 (74%)
Positive	9 (26%)
Local recurrence (46)	
No	31 (67%)
Yes	15 (33%)
Late metastatic disease (44)	
No	24 (55%)
Yes	20 (45%)
Status at last followup (46)	
AWOD	23 (50%)
AWED	6 (13%)
DOD	16 (35%)
DWOD	1 (2%)

AWOD: alive without disease.

AWED: alive with evidence of disease.

DOD: died of disease.

DWOD: died without evidence of disease.

^†^: number of patients with sufficient data for each data point.

^*δ*^: local radiation either as primary local treatment of neoadjuvant.

**Table 2:** Data for patients with single-site metastatic disease.

Patient	Primary location	Metastatic location	Treatment to metastatic location	Outcome at latest followup
1	Femur	Lung	Radiation	DWOD^*δ*^
2	Femur	Lung	Radiation	AWOD
3	Femur	Lung	None	AWOD
4	Clavicle	Lung	None	DOD^†^
5	Femur	Lung/mediastinum	Thoracotomy	AWOD
6	Ilium	Lung	Radiation	AWOD

AWOD: alive without disease.

AWED: alive with evidence of disease.

DOD: died of disease.

DWOD: died without evidence of disease.

^*δ*^: Patient died as result of radiation lung injury.

^†^: Patient died at greater than six years from diagnosis.

**Table 3:** Data for patients with pelvic location EWS.

Patient	Location	Chemotherapy	Radiation	Surgery	Margin	Local recurrence	Outcome
1	Right hemipelvis	Insufficient data	Insufficient data	Right internal hemipelvectomy	Negative	None	Died of disease
2	Right superior ramus/ischium	Yes	Yes	Excision superior ramus	Positive	Yes	Died of disease
3	Sacral/S5	Yes	No	Resection S3–5, coccyx	Negative	None	Alive without disease
4	Right superior ramus	Yes	No	Right internal hemipelvectomy	Negative	None	Alive without disease
5	Right ilium	Yes	No	Right internal hemipelvectomy	Negative	None	Alive without disease
6	Left ilium	Yes	Yes	Left internal hemipelvectomy for recurrence after radiation	Positive	Yes	Died of disease
7	Sacral/S1-S2	No	No	Laminectomy S1-S2/resection tumor	Positive	None	Alive with evidence of disease
8	Right ilium	Yes	Yes	Insufficient data	Insufficient data	None	Alive without disease
9	Right ilium	Yes	Yes	Insufficient data	Insufficient data	None	Died of disease
10	Pelvis (ST/ovary)	No	No	TAH-BSO at OSH	Positive	Yes	Died of disease
11	Right ilium	Yes	No	Right internal hemipelvectomy	Negative	None	Alive without disease

ST: soft tissue EWS.

**Table 4:** Data for patients treated with radiation for sole local control or neoadjuvant therapy prior to surgical resection.

Patient	Primary location	Year of diagnosis	Metastatic disease at diagnosis	Surgical resection after XRT	Recurrence (year)	Complication	Radiation-induced sarcoma
1	Femur	1981	None	Yes/for recurrence	Yes (1983)	None	None
2	Femur	1983	Lung	None	None	Radiation necrosis femur	None
3	Deltoid (ST)	1985	None	Yes/forequarter amputation for recurrence	Yes (1986)	None	None
4	Ischium/ramus	1998	Multicentric	Yes	Yes (1999)	None	None
5	ilium	2000	None	None	None	None	Yes
6	ilium	2001	None	None	None	None	None
7	Thoracic/ mediastinum	2001	None	None	None	Radiation esophagitis	None
8	Femur	2005	Lung/mediastinum	Yes	None	None	None
9	Tibia	2006	Mulitcentric	None	None	None	None
10	Ilium	2007	None	Yes/for recurrence	Yes (2009)	None	None

ST: soft tissue EWS.

**Table 5:** Data for patients with local recurrence.

Patient	Location	Local control	Time till recurrence (years)^†^	Treatment for recurrence	Outcome (at latest followup)
1	2nd Metatarsal	Surgery	2.3	Ifosfamide, Carboplatinum, Etoposide	Alive with evidence of disease
2	Chest wall	Surgery	6	Resection	Died of disease
3	Deltoid (ST)	Radiation	1.2	Forequarter amputation	Died of disease
4	Femur	Surgery	3.1	Reinduction chemotherapy/Distal femur resection	Died of disease
5	Chest wall	Surgery/(initial surgery: incomplete resection at OSH)/then chest wall resection for increasing size while on chemotherapy	0.7	None	Died of disease
6	Chest wall	Surgery	1.2	Resection/Cytoxan/Topotecan	Died of disease
7	Pelvic/ovary (ST)	Surgery	0.1	None	Died of disease
8	Thigh	Surgery	0.2	Resection/Vincristine/Doxorubicin/ Cyclophosphamide	Died of disease
9	Clavicle	Surgery	2.4	Resection/Ifosfamide/Etoposide/ Cytoxan/Topotecan	Alive with evidence of disease
10	Femur	Surgery	0.9	None	Died of disease
11	Popliteal (ST)	Surgery	2.8	Above knee amputation	Alive with evidence of disease
12	Pelvis	Radiation/surgery	1	None	Died of disease
13	Femur	Radiation	2.2	Proximal femur resection	Alive without evidence of disease
14	Pelvis	Radiation	1.4	Hemipelvectomy/Cytoxan/Topotecan/XRT	Died of disease
15	Humerus	Unknown	2.1	Shoulder disarticulation	Alive with evidence of disease

^†^: Time to recurrence calculated from date of diagnosis.

ST: soft tissue Ewing's.

**Table 6:** Univariate analyses of variables for Event free survival (EFS).

Univariate event free survival analysis^*δ*^		
Variable	5-year cumulative survival	*P* Value
Age (46 patients)		0.021^†^
<14	0.78 ± .14	
≥14	0.46 ± .09	
Location (46 patients)		0.164
Appendicular	0.57 ± .10	
Axial	0.45 ± .12	
Pelvic location (46 patients)		0.796
No	0.54 ± .09	
Yes	0.48 ± .16	
Metastatic disease (42 patients)		0.150
No	0.61 ± .09	
Yes	0.29 ± .15	
Multicentric metastatic disease (42 patients)		0.002^†^
No	0.61 ± .09	
Yes	0.00 ± .00	
Bone or soft tissue (46 patients)		0.020^†^
Bone	0.56 ± .08	
Soft tissue	0.29 ± .17	
Local radiation (43 patients)		0.675
No	0.54 ± 0.09	
Yes	0.40 ± 0.16	
Margins (35 patients)		0.337
Negative	0.60 ± .10	
Positive	0.28 ± .21	

^*δ*^: Log Rank of Kaplan Meier EFS survival curves.

^†^: Statistically significant factor.

**Table 7:** Determination of independent variables for Event Free Survival (EFS) with multivariate Cox regression analysis.

Multivariate Cox Regression EFS analysis of covariates			
Variable	HR	95% CI	*P* value
Age			0.638
<14	1.00		
≥14	1.56	0.26–8.56	
Location			0.165
Appendicular	1.00		
Axial	2.22	0.72–6.85	
Multicentric metastatic disease			0.007^†^
No	1.00		
Yes	4.16	1.47–11.81	
Bone or soft tissue			0.014^†^
Bone	1.00		
Soft tissue	5.19	1.40–19.23	
Local radiation			0.397
No	1.00		
Yes	1.55	0.56–4.27	

^†^: statistically significant independent variable on event free survival.

**Table 8:** Univariate analyses of variables for Overall Survival (OS).

Univariate Overall Survival Analysis^*δ*^		
Variable	5-year cumulative survival	*P* Value
Age (46 patients)		0.217
<14	0.74 ± .16	
≥14	0.67 ± .08	
Location (46 patients)		0.308
Appendicular	0.67 ± .10	
Axial	0.68 ± .11	
Pelvic Location (46 patients)		0.455
No	0.69 ± .09	
Yes	0.64 ± .15	
Bone or Soft Tissue (46 patients)		0.066
Bone	0.72 ± .08	
Soft Tissue	0.43 ± .19	
Metastatic Disease (42 patients)		0.061
No	0.70 ± .09	
Yes	0.51 ± .15	
Multicentric Metastatic Disease (42 patients)		0.004^†^
No	0.73 ± .08	
Yes	0.21 ± .18	
Local Radiation (43 patients)		0.398
No	0.70 ± .09	
Yes	0.50 ± .16	
Recurrence (46 patients)		0.001^†^
No	0.83 ± .07	
Yes	0.34 ± .14	
Margins (35 patients)		0.291
Negative	0.75 ± .09	
Positive	0.53 ± .17	

^*δ*^: Log Rank of Kaplan Meier OS survival curves.

^†^: Statistically significant factor.

**Table 9:** Determination of independent variables for Overall Survival (OS) with multivariate Cox regression analysis.

Multivariate Cox Regression OS analysis of covariates			
Variable	HR	95% CI	*P* value
Age			0.8703
<14	1.00		
≥14	0.87	0.16–4.63	
Metastatic disease			0.6000
No	1.00		
Yes	1.57	0.29–8.35	
Multicentric-metastatic disease			0.0291^†^
No	1.00		
Yes	8.23	1.24–54.64	
Bone or soft tissue			0.2187
Bone	1.00		
Soft tissue	2.33	0.61–8.95	
Local recurrence			0.0002^†^
No	1.00		
Yes	11.64	3.24–41.74	

^†^: statistically significant independent variable on overall survival.

## References

[1] Sluga M, Windhager R, Lang S (2001). The role of surgery and resection margins in the treatment of Ewing's sarcoma. *Clinical Orthopaedics and Related Research*.

[2] Rodriguez-Galindo C, Spunt SL, Pappo AS (2003). Treatment of ewing sarcoma family of tumors: current status and outlook for the future. *Medical and Pediatric Oncology*.

[3] Rosito P, Mancini AF, Rondelli R (1999). Italian cooperative study for the treatment of children and young adults with localized ewing sarcoma of bone: a preliminary report of 6 years of experience. *Cancer*.

[4] Delattre O, Zucman J, Melot T (1994). The Ewing family of tumors—a subgroup of small-round-cell tumors defined by specific chimeric transcripts. *New England Journal of Medicine*.

[5] Ludwig JA (2008). Ewing sarcoma: historical perspectives, current state-of-the-art, and opportunities for targeted therapy in the future. *Current Opinion in Oncology*.

[6] Herzog CE (2005). Overview of sarcomas in the adolescent and young adult population. *Journal of Pediatric Hematology/Oncology*.

[7] Maheshwari AV, Cheng EY (2010). Ewing sarcoma family of tumors. *Journal of the American Academy of Orthopaedic Surgeons*.

[8] Rodríguez-Galindo C, Navid F, Liu T, Billups CA, Rao BN, Krasin MJ (2008). Prognostic factors for local and distant control in Ewing sarcoma family of tumors. *Annals of Oncology*.

[9] Rodríguez-Galindo C, Liu T, Krasin MJ (2007). Analysis of prognostic factors in Ewing sarcoma family of tumors. *Cancer*.

[10] Cotterill S, Ahrens S, Paulussen M (2000). Prognostic factors in Ewing's tumor of bone: analysis of 975 patients from the European Intergroup Cooperative Ewing's Sarcoma Study Group. *Journal of Clinical Oncology*.

[11] Jürgens H, Exner U, Gadner H (1988). Multidisciplinary treatment of primary Ewing's sarcoma of bone: a 6-year experience of a European Cooperative trial. *Cancer*.

[12] Lee J, Hoang BH, Ziogas A, Zell JA (2010). Analysis of prognostic factors in Ewing sarcoma using a population-based cancer registry. *Cancer*.

[13] Bacci G, Ferrari S, Bertoni F (2000). Prognostic factors in nonmetastatic Ewing's sarcoma of bone treated with adjuvant chemotherapy: analysis of 359 patients at the Istituto Ortopedico Rizzoli. *Journal of Clinical Oncology*.

[14] Craft A, Cotterill S, Malcolm A (1998). Ifosfamide-containing chemotherapy in Ewing's sarcoma: the second United Kingdom Children's Cancer Study Group and the Medical Research Council Ewing's Tumor Study. *Journal of Clinical Oncology*.

[15] Esiashvili N, Goodman M, Marcus R (2008). Changes in incidence and survival of ewing sarcoma patients over the past 3 decades. *Journal of Pediatric Hematology/Oncology*.

[16] Miser JS, Krailo MD, Tarbell NJ (2004). Treatment of metastatic Ewing's sarcoma or primitive neuroectodermal tumor of bone: evaluation of combination ifosfamide and etoposide—a children's cancer group and pediatric oncology group study. *Journal of Clinical Oncology*.

[17] Bacci G, Toni A, Avella M (1989). Long-term results in 144 localized Ewing's sarcoma patients treated with combined therapy. *Cancer*.

[18] Capanna R, Toni A, Sudanese A, McDonald D, Bacci G, Campanacci M (1990). Ewing's sarcoma of the pelvis. *International Orthopaedics*.

[19] Li WK, Lane JM, Rosen G (1983). Pelvic Ewing's sarcoma. Advances in treatment. *Journal of Bone and Joint Surgery*.

[20] Frassica FJ, Frassica AD, Pritchard DJ, Schomberg PJ, Wold LE, Sim FH (1993). Ewing sarcoma of the pelvis. Clinicopathological features and treatment. *Journal of Bone and Joint Surgery*.

[21] Ahmad R, Mayol BR, Davis M, Rougraff BT (1999). Extraskeletal Ewing's sarcoma. *Cancer*.

[22] Burgert EO, Nesbit ME, Garnsey LA (1990). Multimodal therapy for the management of nonpelvic, localized Ewing's sarcoma of bone: intergroup study IESS-II. *Journal of Clinical Oncology*.

[23] Shankar AG, Pinkerton CR, Atra A (1999). Local therapy and other factors influencing site of relapse in patients with localised Ewing's sarcoma. *European Journal of Cancer*.

[24] Yang RS, Eckardt JJ, Eilber FR (1995). Surgical indications for Ewing's sarcoma of the pelvis. *Cancer*.

[25] Wunder JS, Paulian G, Huvos AG, Heller G, Meyers PA, Healey JH (1998). The histological response to chemotherapy as a predictor of the oncological outcome of operative treatment of Ewing Sarcoma. *Journal of Bone and Joint Surgery*.

[26] Hayes FA, Thompson EI, Meyer WH (1989). Therapy for localized Ewing's sarcoma of bone. *Journal of Clinical Oncology*.

[27] Pradhan A, Grimer RJ, Spooner D (2011). Oncological outcomes of patients with Ewing's sarcoma: is there a difference between skeletal and extraskeletal ewing's sarcoma?. *Journal of Bone and Joint Surgery*.

[28] Rodriguez-Galindo C, Billups CA, Kun LE (2002). Survival after recurrence of ewing tumors. *Cancer*.

[29] Rud NP, Reiman HM, Pritchard DJ, Frassica FJ, Smithson WA (1989). Extraosseous Ewing's sarcoma. *Cancer*.

[30] Nesbit ME, Gehan EA, Burgert EO (1990). Multimodal therapy for the management of primary, nonmetastatic Ewing's Sarcoma of bone: a long-term follow-up of the first intergroup study. *Journal of Clinical Oncology*.

[31] Picci P, Böhling T, Bacci S (1997). Chemotherapy-induced tumor necrosis as a prognostic factor in localized Ewing's sarcoma of the extremities. *Journal of Clinical Oncology*.

[32] Bacci G, Picci P, Gitelis S (1982). The treatment of localized Ewing's sarcoma: the experience at the Istituto Ortopedico Rizzoli in 163 cases treated with and without adjuvant chemotherapy. *Cancer*.

[33] Applebaum MA, Worch J, Matthay KK (2011). Clinical features and outcomes in patients with extraskeletal Ewing sarcoma. *Cancer*.

